# Obesity-associated insulin resistance adversely affects skin function

**DOI:** 10.1371/journal.pone.0223528

**Published:** 2019-10-03

**Authors:** Masafumi Aoki, Takatoshi Murase

**Affiliations:** Biological Science Laboratories, Kao Corporation, Ichikai-machi, Haga-gun, Tochigi, Japan; Medical University of Vienna, AUSTRIA

## Abstract

The aim of this study was to identify changes in skin function associated with obesity and the mechanisms underlying these changes. Functional changes and gene expression in skin were investigated in C57BL/6J mice fed either a control or high-fat diet (HFD). The insulin responsiveness of the skin and skeletal muscle was also evaluated. The effects of inhibiting insulin signaling and altered glucose concentration on skin function-associated molecules and barrier function were analyzed in keratinocytes. HFD-fed mice were not only severely obese, but also exhibited impaired skin barrier function and diminished levels of glycerol transporter aquaporin-3, keratins, and desmosomal proteins involved in maintaining skin structure. Moreover, the expression of cell cycle regulatory molecules was altered. Insulin signaling was attenuated in the skin and skeletal muscle of HFD-fed mice. In keratinocytes, inhibition of insulin signaling leads to decreased keratin expression and diminished barrier function, and higher glucose concentrations increased the expression of CDK inhibitor 1A and 1C, which are associated with cell-cycle arrest. Obesity-associated impairment of skin function can be attributed to structural fragility, abnormal glycerol transport, and dysregulated proliferation of epidermal cells. These alterations are at least partly due to cutaneous insulin resistance and hyperglycemia.

## Introduction

Obesity is a key risk factor for type 2 diabetes, hypertension, and cardiovascular disease [1–3], conditions associated with insulin resistance. Increased production of tumor necrosis factor α (TNFα) and non-esterified fatty acids (NEFAs) by hypertrophied adipocytes leads to reduced levels and dysregulation of insulin signaling molecules such as insulin receptor substrate (IRS), resulting in insulin resistance in the liver and skeletal muscle [4]. The heightened oxidative stress and inflammation associated with obesity are also involved in the onset and exacerbation of insulin resistance [4, 5]. Meeolic abnormalities induced by insulin resistance are widely recognized to relate to disorders such as type 2 diabetes and hypertension.

The skin, consisting of the epidermis, dermis, and subcutaneous tissue, is the largest organ in the body; through its barrier function, it plays crucial roles in protecting the body against the external environment and in maintaining internal conditions [6–8]. In particular, the stratum corneum barrier, composed of corneocytes and intercellular lipids, protects the body against adverse factors such as microorganisms, chemicals, antigens, and ultraviolet light, as well as against excessive water loss. Failure of this function is considered to confer greater susceptibility to various stimuli and allergens, thereby easily giving rise to inflammation and itch. The barrier function is therefore crucial for maintaining skin homeostasis, and is considered an indicator of cutaneous health [9].

Epidermal cells express keratin cytoskeletal proteins and are connected with neighboring cells by desmosomes, forming a robust protective structure [10, 11]. Keratin 1 (KRT1), KRT5, KRT10, and KRT14 are mainly expressed in the epidermis. Abnormalities of these molecules cause skin fragility diseases, such as epidermolysis bullosa simplex and epidermolytic/bullous ichthyosis [12, 13]. In addition, mice lacking keratin genes usually exhibit fragile skin or a lethal phenotype [14]. Desmosomes are intercellular junctions composed of multiple molecules, including desmoglein (DSG), desmocollin (DSC), plakophilin (PKP), desmoplakin (DSP), and junction plakoglobin (JUP) [15]. Mutations or absence of desmosomal genes cause a lethal phenotype or skin fragility [15, 16]. In the stratum corneum, the keratinocyte proliferation/differentiation balance and lipid homeostasis (e.g., ceramide, cholesterol, and glycerol metabolism) are also important for maintaining skin barrier function [11, 17, 18].

Obesity is a leading cause of not only systemic metabolic abnormalities such as glucose and lipid metabolism disorders, but also increased oxidative stress and inflammation [19]. Obesity -associated increases in inflammatory cytokines and oxidative stress are speculated to contribute to various skin impairments [20–22]. Some studies demonstrated that obesity is accompanied by diminished skin barrier function, dry skin, and itching, which affects quality of life [23–25]. In addition, we recently characterized the skin function of obese and non-obese subjects, and identified possible factors influencing impaired skin function, including altered autonomic nerve-vascular system activity, inflammation and insulin resistance [26]. Moreover, obesity and diabetes adversely affect the skin [27]. For example, inflammatory cell infiltration [28], reduction of epidermal thickness [29], imbalance of cellular differentiation/proliferation [30, 31], and barrier dysfunction [32] are present in the skin of diabetic animal models or humans diagnosed with diabetes. The mechanisms underlying obesity-associated skin impairment, however, remain unclear. Therefore, this study aimed to determine the molecular events involved in skin impairment caused by diet-induced obesity.

## Materials and methods

### Animals and diets

Male, 7-week-old C57BL/6J mice were purchased from Japan SLC (Shizuoka, Japan), and maintained under a 12-h:12-h light:dark cycle (with lights on from 7 AM to 7 PM) at 23 ± 2°C. Mice were divided into two groups (N = 20, 5 mice/cage) and allowed ad libitum access to water and either control feed (41.8 kJ% fat) (D12450B; Research Diets, New Brunswick, NJ) or high-fat feed (high-fat diet [HFD]; 251.0 kJ% fat) (D12492; Research Diets) for 26 weeks. Mouse health status was monitored daily and body weight was measured once per week. All animal experiments were approved by the Animal Care Committee of Kao Tochigi Institute (approval number: N2011-0063A).

### Blood analysis and organ sampling

After 26 weeks on control or high-fat diet, animals were fasted for 3 h. Mice were euthanized by isoflurane overdose, and blood was collected via the postcaval vein. Organs from each mouse were resected and weighed. Serum was separated from the whole blood, and triglyceride, total cholesterol, high-density lipoprotein cholesterol, and low-density lipoprotein cholesterol concentrations, and glutamic oxaloacetic transaminase and glutamic pyruvic transaminase activity were determined using the following enzyme-based assay kits (N-assays; Nittobo Medical, Tokyo, Japan): L TG-H, L T-CHO-H, L HDL, L LDL-S, L GOT, and L GPT, respectively. NEFA levels were measured using a NEFA-HA test (Wako Pure Chemical Industries, Osaka, Japan). Total adiponectin, high-molecular-weight adiponectin, interleukin (IL) -1α, IL-1β, TNFα, lipid peroxidation (thiobarbituric acid reactive substances), and total antioxidant capacity were measured using enzyme-linked immunosorbent assay kits for mouse adiponectin (Assaypro, St. Charles, MO), mouse/rat high-molecular-weight adiponectin (Shibayagi, Gunma, Japan), mouse IL-1α (BioVendor, Brno, Czech Republic), mouse IL-1β (RayBiotech, Norcross, GA), and mouse TNF-α (Quantikine; R&D Systems, Minneapolis, MN), a TBARS Colorimetric Microplate Assay kit (Oxford Biomedical Research, Oxford, MI), and an OxiSelect Total Antioxidant Capacity Assay kit (Cell Biolabs, San Diego, CA), according to the manufacturers’ instructions. Diacron reactive oxygen metabolites and biological antioxidant potential were measured with a free radical analyzer system (FREE Carpe Diem; Wismerll, Tokyo, Japan).

### Oral glucose tolerance test

After 25 weeks of feeding, an oral glucose tolerance test (OGTT) was performed according to the method of Jiang *et al*. [33]. Glucose (2 g/kg body weight) was orally administered to mice (N = 7) after overnight fasting, and blood samples were collected from the orbital sinus at 0, 15, 30, 60, and 120 min. Blood glucose concentrations were measured with a portable blood glucose meter (Accu-Chek Aviva; Roche Diagnostics, Mannheim, Germany), and serum insulin levels were measured with a mouse insulin enzyme-linked immunosorbent assay kit (Morinaga, Kanagawa, Japan).

### Histologic analysis

Inguinal subcutaneous adipose tissue soaked in 4% paraformaldehyde was embedded in paraffin and cut into thin sections. The sections were mounted on slides, deparaffinized in xylene, and stained with an anti-F4/80 antibody (Novus Biologicals, Littleton, CO). After incubation with Histofine Simple Stain MAX-PO (Nichirei, Tokyo, Japan), color was developed with 3,3′-diaminobenzidine tetrahydrochloride. Sections were rinsed and counterstained with Mayer’s hematoxylin. Skin samples were collected and fixed with 4% paraformaldehyde, embedded in paraffin, and cut into thin sections. Paraffin sections were analyzed by hematoxylin and eosin (H/E) staining.

### Skin function measurements

After 25 weeks of feeding, the mice were anesthetized with an intraperitoneal injection pentobarbital (Somnopentyl; Kyoritsu Seiyaku, Tokyo, Japan), the backs of the mice (N = 8) were shaved, and transepidermal water loss (TEWL) and skin capacitance were measured with a Tewameter TM 300 (Courage + Khazaka Electronic GmbH, Cologne, Germany) and a Corneometer CM 825 (Courage + Khazaka Electronic GmbH), respectively.

### Determination of tissue insulin response

After 25 weeks of feeding, the insulin sensitivity of the skeletal muscle and skin of the mice was measured according to the method of Zong *et al*. [34]. Mice were fasted for 14 h and administered an intraperitoneal injection of recombinant insulin (1 U/kg body weight; Sigma, St. Louis, MO). Ten minutes later, gastrocnemius muscle and skin samples were collected and subjected to western blotting with anti-Akt, anti-phospho-Akt (Ser473), anti-p70S6K, and anti-phospho-p70S6K (Thr389) antibodies (Cell Signaling, Danvers, MA), as described below. Skin was collected from the shaved backs of the mice; after removal of the subcutaneous tissue and fascia, skin samples including both the dermis and epidermis were used for analyses.

### Preparation of viruses to decrease insulin receptor expression

Viral vectors encoding insulin receptor-silencing short hairpin RNA (IR shRNA) were prepared with the ViraPower adenoviral expression system (Thermo Fisher Scientific, Waltham, MA). Three vectors were constructed, each carrying a different IR shRNA sequence expressed under control of the human U6 promoter (GenBank accession No. X07425). These IR shRNA-encoding DNA sequences are shown in S1 Table. A virus carrying the β-galactosidase gene *lacZ* was used as a control.

### Effects of insulin signaling inhibition on three-dimensional keratinocyte cultures (3D-keratinocytes)

An *in vitro* model of the human epidermis (LabCyte EPI-MODEL) and assay medium were obtained from J-TEC (Gamagori, Japan), and cells were cultured in an atmosphere of 95% air and 5% CO_2_ at 37°C. On day 2 of culture, insulin signaling was inhibited with the insulin signaling inhibitor wortmannin (Cell Signaling) or diminished through transduction with the IR shRNA virus (25 PFU/cell MOI). Cells receiving the former treatment were administered 2 μmol/l wortmannin and cultured for 3 days, whereas those receiving the latter treatment were exposed to IR shRNA virus stock solution for 14 h, and then incubated for 3 days in assay medium. At the end of the experiment, the cells were washed with PBS (-) (Thermo Fisher Scientific), and samples were collected for gene and protein expression analysis.

### Effects of insulin signaling inhibition on barrier function of 3D-keratinocytes

After inhibiting insulin signaling in 3D-keratinocytes with wortmannin, barrier function was assessed by Lucifer yellow permeability assays using the method of Pendaries *et al*. [35]. Briefly, 200 μl of 1mM Lucifer yellow (Wako) was added onto the 3D-keratinocytes treated with wortmannin (2 μmol/l) for 3 days, and then incubated at 37°C for 6 h. The cells were fixed in 4% formaldehyde and embedded in paraffin. Sections were imaged with a fluorescence microscope (BZ-X710, Keyence, Osaka, Japan). Lucifer yellow penetration into 3D-keratinocytes was measured using a BZ-X Analyzer (Keyence).

### Effects of glucose concentration on keratinocytes

Neonatal human epidermal keratinocytes (HEKn; Thermo Fisher Scientific) were cultured in EpiLife medium (Thermo Fisher Scientific) supplemented with HuMedia-KG (Kurabo, Osaka, Japan) and grown to subconfluence. The culture medium was then replaced with EpiLife medium containing 6, 10, 20, or 40 mmol/l glucose, and the cells were cultured for an additional 3 days. After the cells were washed with PBS (-), samples were collected for further analyses.

### RNA extraction and quantitative real-time RT-PCR

Total RNA was isolated from mouse tissue using Isogen reagent (Wako Pure Chemical Industries) and from 3D-keratinocytes and HEKn cells using an RNeasy Mini Kit (Qiagen, Hilden, Germany), then reverse-transcribed using a High-Capacity RNA-to-cDNA kit (Thermo Fisher Scientific). Quantitative real time polymerase chain reaction (RT-qPCR) was performed using Fast SYBR Green Master Mix (Thermo Fisher Scientific) or TaqMan Fast Universal PCR Master Mix (Thermo Fisher Scientific) on a 7500 Fast Real-Time PCR System (Thermo Fisher Scientific). The following mouse genes were analyzed using TaqMan Gene Expression Assays (Thermo Fisher Scientific): *Dsg1a* (Mm00809994_s1), *Dsg1b* (Mm00839130_mH), and *Dsg1c* (Mm00725121_g1). Details of the primer pairs used with Fast SYBR Green Master Mix are given in S2 Table.

### Western blot analysis

Mouse tissue (frozen whole skin and muscle samples) and 3D-keratinocytes were homogenized and sonicated in CelLytic MT Mammalian Tissue Lysis/Extraction Reagent (Sigma), and then centrifuged at 16,000*g* for 10 min at 4°C. The supernatants were harvested, and equal amounts of protein were subjected to SDS-PAGE and transferred to polyvinylidene fluoride membranes. The membranes were then incubated with primary antibodies to KRT5 (Abcam, Cambridge, UK), KRT10 (Abcam), KRT14 (Santa Cruz Biotechnology, Dallas, TX), desmoglein 1 (DSG1; GeneTex, San Antonio, TX), DSG2 (Abcam), desmocollin 3 (DSC3; Santa Cruz Biotechnology), α-tubulin (Cell Signaling), Akt (Cell Signaling), phospho-Akt (Ser473) (Cell Signaling), p70S6K (Cell Signaling), and phospho-p70S6K (Thr389) (Cell Signaling). After washing the membranes with 0.1% Tween 20 in PBS, they were exposed to a horseradish peroxidase-linked anti-rabbit IgG antibody (Cell Signaling). Bands were visualized with Amersham ECL Prime Western Blotting Detection Reagent (GE Healthcare, Little Chalfont, UK) and their intensities were quantified using the program Multi Gauge (Fujifilm, Tokyo, Japan).

### Statistical analysis

All values are reported as means ± SD. Unpaired *t*-tests were used for comparisons between the control and HFD-fed groups in the animal experiments, and between the control and wortmannin groups, and the *lac*Z and IR shRNA groups in the 3D-keratinocyte experiments. For the HEKn cell experiments, each group was compared with the control group using analysis of variance followed by Dunnett’s test. These analyses were carried out with GraphPad Prism 6 (GraphPad Software, La Jolla, CA). A *p* value < 0.05 was considered statistically significant.

## Results

### Changes in mouse body weight, body composition, and serum components

Compared with mice fed the control diet, mice fed the HFD for 26 weeks had significantly higher body and adipose tissue weights (S3 Table). Moreover, compared with the control group, HFD-fed mice had altered levels of serum components generally associated with obesity, such as hyperlipidemia, lower adiponectin levels, increased inflammatory cytokine levels, and greater levels of oxidative stress markers (S3 Table).

### Changes in glucose metabolism and subcutaneous adipose tissue inflammation associated with obesity

To investigate the metabolic changes that occurred after 25 weeks on the HFD diet, we performed an OGTT analysis. Blood glucose concentrations were significantly higher in the HFD group than in the control group at 15 and 30 min after glucose administration (S1A Fig). Insulin levels were also significantly higher in the HFD group compared with the control group at all time-points, suggesting that glucose tolerance was impaired in HFD-fed mice (S1B Fig). We next examined the inflammatory response in subcutaneous adipose tissue associated with obesity. mRNA expression of genes encoding the macrophage marker F4/80 and monocyte- and lymphocyte-recruiting chemokine monocyte chemoattractant protein-1 (MCP-1) was significantly higher (2.4-fold) in the subcutaneous adipose tissue of HFD-fed mice than in mice fed the control diet (S1C Fig). Moreover, staining with an antibody to F4/80 revealed that macrophages were highly prevalent in the subcutaneous adipose tissue of the HFD-fed mice (S1D Fig), indicating increased inflammation.

### Changes in skin function associated with obesity

**Fig 1:**
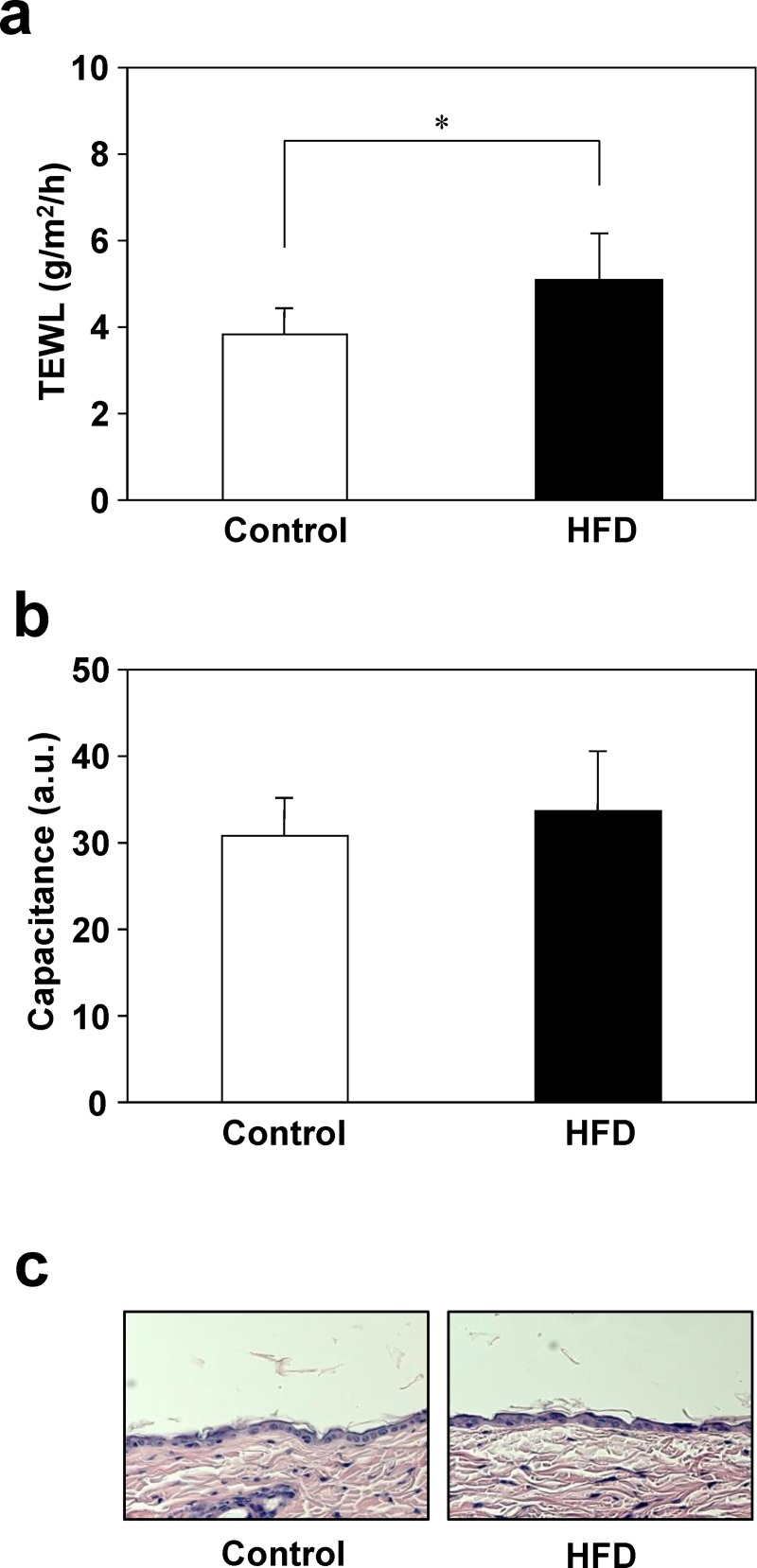
Skin function in mice fed the control diet or HFD. After 25 weeks of feeding, the dorsal skin of control and HFD-fed mice was shaved and TEWL (a) and skin capacitance (b) were measured using a Tewameter TM 300 and Corneometer CM 825, respectively. Dorsal skin was collected from mice fed control or HFD for 26 weeks, and were stained with H/E (c). Values are means ± SD (N = 8). *p < 0.05 vs. the control group (Student’s *t*-test). a.u., arbitrary units.

To evaluate the obesity-associated changes in skin function, we measured the TEWL and capacitance, measures of skin barrier function and hydration, respectively, after 25 weeks of feeding. The TEWL was 33% higher in the HFD group than in the control group, indicating that HFD-fed mice had impaired barrier function (Fig 1A). In contrast, there was no difference in skin capacitance between groups (Fig 1B). We detected few histological differences in the skin from mice fed control or HFD (Fig 1C).

### Gene and protein expression in the skin

**Fig 2:**
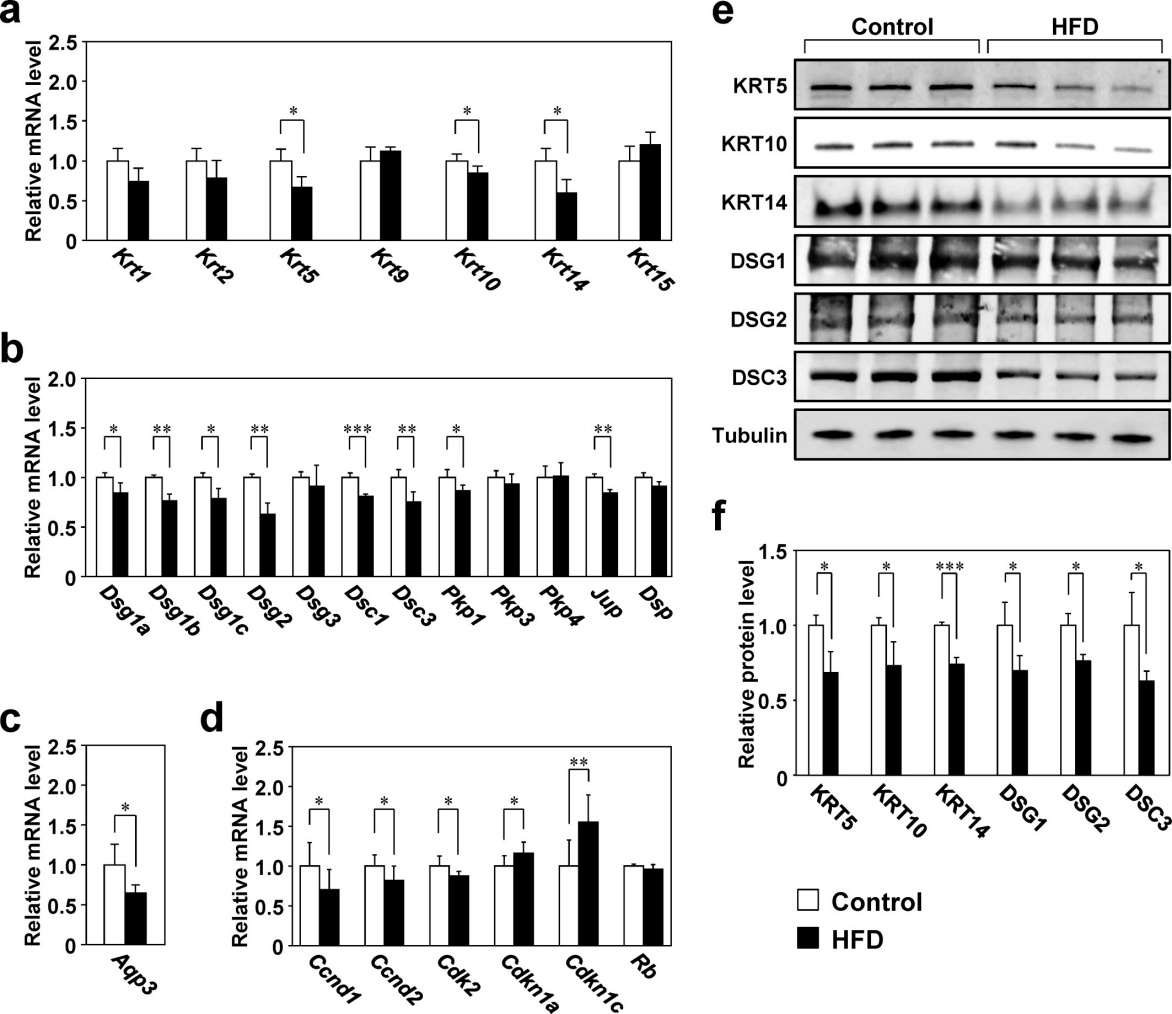
Gene and protein expression in the skin of mice fed the control diet or HFD. a–d: RNA was isolated from the back skin of mice fed the control diet or HFD for 26 weeks, and gene expression was analyzed by RT-qPCR. The expression level of each target gene was normalized to that of *36B4* and is shown relative to that of the control group. Values are means ± SD (N = 8). *p < 0.05, **p < 0.01, and ***p < 0.001 vs. the control group (Student’s *t*-tests).e, f: Total proteins extracted from the dorsal skin were analyzed by western blotting and their levels were normalized to that of α-tubulin. Values are means ± SD (N = 3). *p < 0.05 and ***p < 0.001 vs. the control group (Student’s *t*-tests).

To examine the effect of obesity on skin structural molecules, keratin and desmosome component gene expression was examined by RT-qPCR [11, 15, 36]. mRNA levels of *Krt5*, *Krt10*, and *Krt14*, which are principally expressed in the basal layer and the stratum spinosum of the epidermis, were significantly lower (15%–40%) in the HFD group than in the control group (Fig 2A). Expression of the desmosomal genes *Dsg1a*, *Dsg1b*, *Dsg1c*, *Dsg2*, *Dsc1*, *Dsc3*, *Pkp1*, and *Jup* was also significantly lower (by 40%–50%) in the HFD group than in the control group (Fig 2B). In addition, the protein levels of KRT5, KRT10, KRT14, DSG1, DSG2, and DSC3 were significantly lower (by 24%–37%) in the HFD group than in the control group (Fig 2E and 2F). Aquaporin 3 (*Aqp3*) mRNA expression, a glycerol channel involved in skin barrier function maintenance [37], was significantly lower in the HFD group than in the control group (Fig 2C). To examine alterations of epidermal cell proliferation associated with obesity, expression of cell cycle regulatory genes was examined in the skin of HFD- or control-fed mice [38]. In the HFD group, the mRNA levels of the *Ccnd1*, *Ccnd2*, and *Cdk2*, which are involved in the G1/S transition, were 13%–30% lower, whereas those of *Cdkn1a* and *Cdkn1c*, which are involved in cell cycle arrest, were 16% and 55% higher, respectively, than those in the control group (Fig 2D).

### Insulin resistance in skeletal muscle and skin

**Fig 3:**
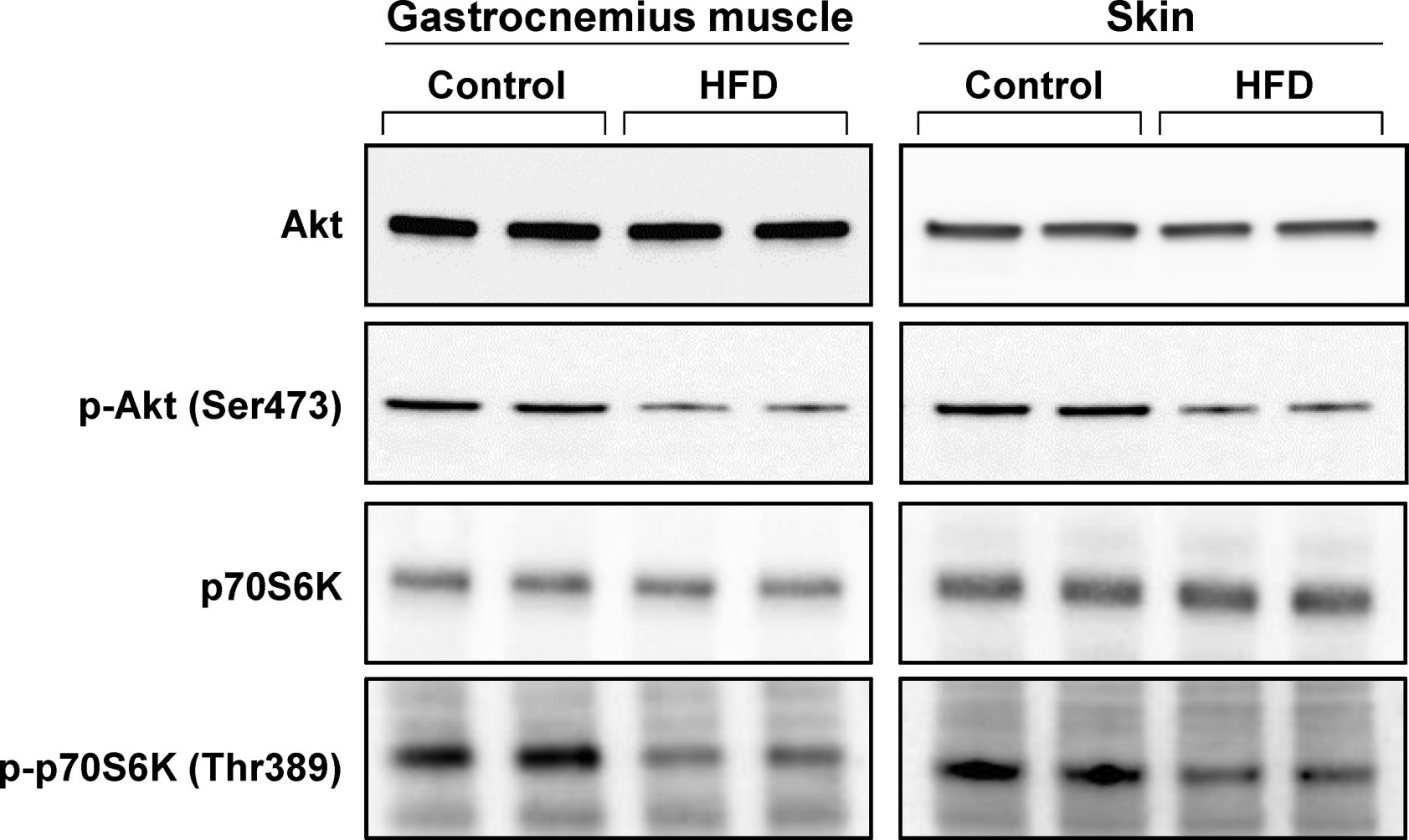
Muscle and skin response to insulin in mice fed the control diet or HFD. At week 25 of the experiment, the control and HFD-fed mice were fasted for 14 h and administered insulin (1 U/kg body weight) intraperitoneally. Ten minutes later, the gastrocnemius muscle and whole skin, including both the dermis and epidermis, were isolated and western blotting was performed with anti-Akt, anti-phospho-Akt (p-Akt), anti-p70S6K, and anti-phospho-p70S6K (p-p70S6K) antibodies.

We next evaluated the insulin responsiveness of the skin of HFD-fed mice by measuring Akt and p70S6K phosphorylation after insulin injection at week 25 of the feeding experiment. Phosphorylation of Akt (Ser473) and p70S6K (Thr389) was decreased in the skin as well as in the skeletal muscle of HFD-fed mice, suggesting that insulin resistance in the skin was induced in association with the development of obesity (Fig 3).

### Effect of insulin signaling inhibition on keratin expression and barrier function in 3D-keratinocytes

**Fig 4:**
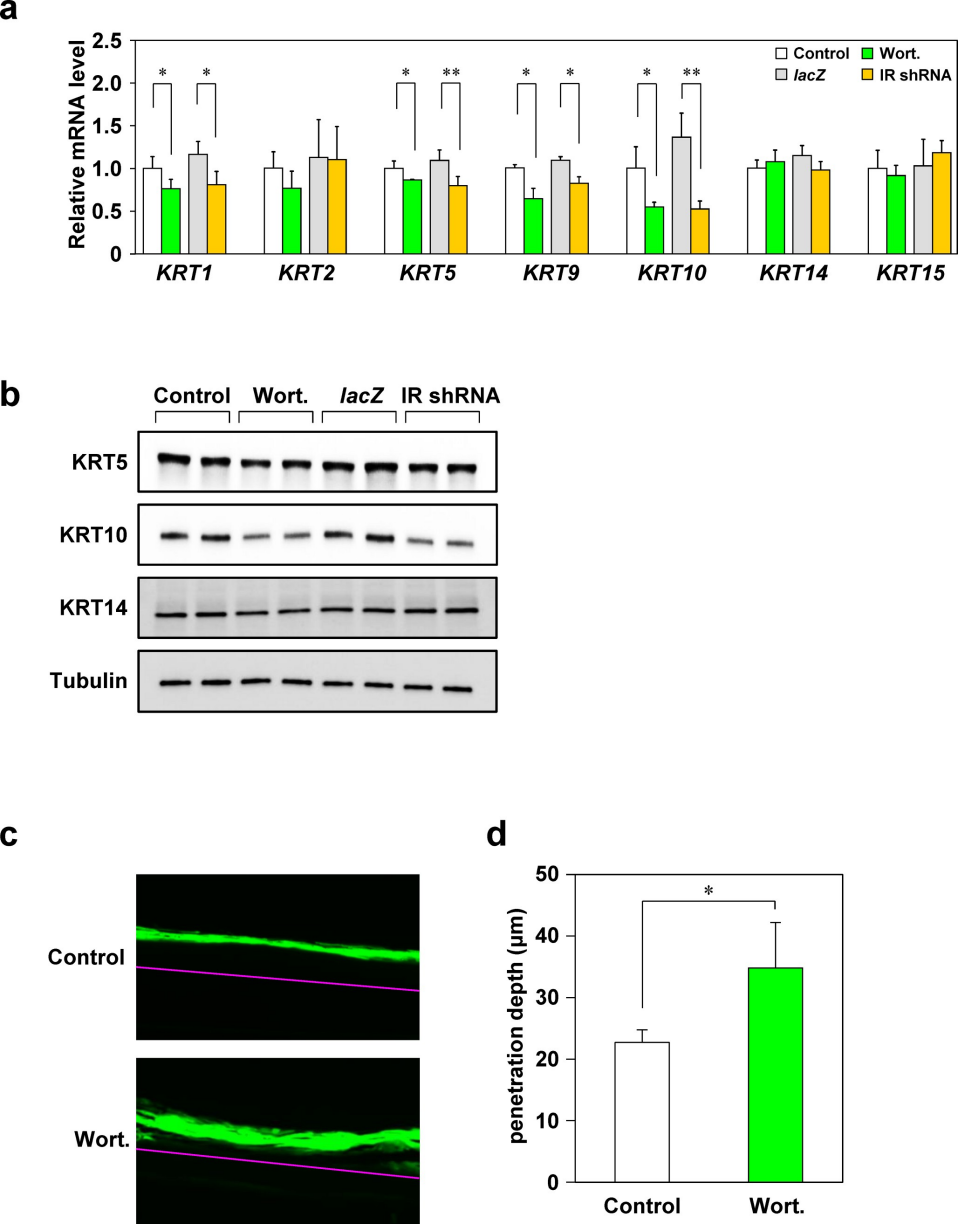
Alteration of keratin expression and barrier function due to insulin signaling inhibition in 3D-keratinocytes. Insulin signaling in 3D-keratinocytes was suppressed by addition of the insulin signaling inhibitor wortmannin (Wort; 2 μM) or transduction with an IR shRNA viral vector. Three days after insulin signaling inhibition, total RNA was isolated, and keratin gene expression was analyzed by RT-qPCR (N = 3). Expression was normalized to that of 36B4 and is shown relative to that of the control group (a). Total protein extracted from cells was analyzed by western blotting (b). Lucifer yellow solution was added onto the 3D-keratinocytes treated with or without wortmannin, and then incubated at 37°C for 6 h. Lucifer yellow penetration into 3D-keratinocytes was investigated using a fluorescence microscope (N = 4). The pink line indicates the boundary between 3D-keratinocytes and membrane filter (c). Dye penetration was measured using a BZ-X Analyzer (d). Values are means ± SD. *p < 0.05 and **p < 0.01 for control vs. Wort or *lac*Z vs. IR shRNA (Student’s *t*-tests).

To determine the effects of insulin resistance on the skin, we inhibited insulin signaling in 3D-keratinocytes and examined the expression of skin function-related molecules whose levels in mice were influenced by the HFD. We inhibited insulin signaling by either treatment with wortmannin or transduction of a virus that suppressed IR expression. First, we confirmed that viral infection attenuated insulin signaling in 3D-keratinocytes (S2 Fig). Inhibition of insulin signaling significantly decreased the levels of *KRT1*, *KRT5*, *KRT9*, and *KRT10* mRNA (by 13%–62%) in 3D-keratinocytes (Fig 4A). Similarly, inhibition of insulin signaling decreased KRT5 and KRT10 protein levels (Fig 4B), indicating that expression of these molecules is directly influenced by insulin resistance. Although KRT14 levels were reduced in the skin of HFD-fed mice, no marked change in the expression of this protein was observed in 3D-keratinocytes upon inhibition of insulin signaling. The expression of desmosomal molecules, cell cycle regulatory molecules, and *AQP3* was not significantly altered (S3 Fig), suggesting that these molecules were affected by factors other than insulin resistance. To further investigate the effect of insulin signaling inhibition on barrier function in 3D-keratinocytes, we performed Lucifer yellow permeability assays. Lucifer yellow penetrated significantly deeper into the stratum corneum of wortmannin-treated 3D-keratinocytes (Fig 4C and 4D).

### Effects of glucose concentration on keratinocyte gene expression

**Fig 5:**
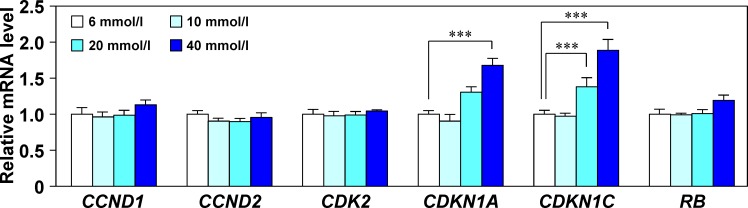
Effects of glucose concentration on the expression of cell cycle regulatory genes. After culture of human epidermal keratinocytes in growth medium containing 6 (control), 10, 20, or 40 mmol/l glucose for 3 days, cell cycle regulatory gene expression levels were analyzed by RT-qPCR. Expression was normalized to that of *36B4* and is shown relative to that of the control group. Values are means ± SD (N = 3). ***p < 0.001 vs. the control group (Dunnett’s test).

Because hyperglycemia is induced by obesity-associated insulin resistance, we investigated whether high glucose concentrations influence the expression of cell cycle regulatory molecules, keratins, desmosomal molecules, and *AQP3*. The mRNA levels of cyclin D1 (*CCND1*), *CCND2*, and cyclin-dependent kinase 2 (*CDK2*) were unchanged by glucose treatment, whereas those of CDK inhibitor 1A (*CDKN1A*) and *CDKN1C* increased in a glucose concentration-dependent manner, suggesting that upregulation of these genes resulted directly from the increased glucose concentration (Fig 5). In contrast, the expression of keratins, desmosomal molecules, and *AQP3* did not change in response to changes in the glucose concentration (S4 Fig).

## Discussion

Insulin resistance is the main pathogenic mechanism underlying type 2 diabetes, hypertension, and cardiovascular disease, and obesity is a major risk factor for these conditions [1–3]. The findings of the present study indicated that obesity strongly affects the skin, and suggest that the insulin resistance associated with obesity is involved in obesity-related skin function impairment. Functional and molecular analyses of the skin of mice with diet-induced obesity and insulin resistance revealed impaired barrier function and concomitant altered expression of keratins and cell cycle regulatory molecules. In addition, disruption of insulin signaling leads to decreased keratin expression and reduced barrier function in 3D-keratinocytes. Further, high glucose concentration retarded HEKn cell cycle progression. These findings suggest that insulin resistance associated with obesity impairs skin function at least in part by inducing structural fragility and disrupting the cell cycle balance in this organ.

Keratins are major cutaneous structural proteins. In the epidermis, KRT5 and KRT14 are expressed in the basal layer, KRT1 and KRT10 are expressed in the lower stratum spinosum, and KRT2 and KRT9 are expressed in the upper stratum spinosum to the stratum granulosum [11, 36, 39]. The desmosomal proteins Dsg and Dsc play important roles in connecting adjacent cells, and are bonded to keratin filaments via Pkp, Jup, and Dsp [15, 40]. In this study, the skin of HFD-fed mice exhibited diminished levels of KRT5, KRT10, KRT14, and a number of desmosomal molecules. Keratin dysfunction leads to various skin diseases. For example, mutations in KRT5 or KRT14 are implicated in epidermolysis bullosa simplex, and KRT1 or KRT10 dysfunction causes bullous congenital ichthyosiform erythroderma [13]; moreover, fragile skin structure is observed in almost all such conditions. In addition, retinoid-induced downregulation of the desmosomal protein DSG1 results in skin fragility [41]. Furthermore, skin barrier disorders are frequently observed in keratin or desmosomal gene-knockout mice [42, 43]. These results suggest that the obesity-related reduction of keratins and desmosome structural components may cause skin fragility and contribute to skin barrier dysfunction.

HFD-fed mice had increased serum levels of inflammatory cytokines and oxidative stress markers, and elevated expression of MCP-1 and macrophage infiltration in subcutaneous adipose tissue. Increased production of TNFα and NEFAs by adipose tissue induces insulin resistance by suppressing tyrosine phosphorylation of IR or IRS-1, leading to abnormal metabolic activity in the liver, skeletal muscle, and adipose tissue [19]. IL-1α, IL-1β, and oxidative stress also induce insulin resistance by modulating IR or IRS-1 phosphorylation [44–46]. On the basis of these previous findings, we hypothesized that insulin resistance is involved in obesity-induced skin dysfunction. In the present work, OGTTs revealed impaired glucose metabolism in HFD-fed mice. In addition, phosphorylation of Akt (Ser473) and p70S6K (Thr389) following injection of insulin were suppressed in the skin as well as the skeletal muscle of such mice, indicating cutaneous insulin resistance. Moreover, not only loss of keratin expression, but also a decrease in barrier function, was observed in 3D-keratinocytes with suppressed insulin signaling. Previous studies demonstrated that the expression of *KRT1* during Ca^2+^-induced differentiation of keratinocytes is upregulated in the presence of insulin [47], and induction of KRT1 and KRT10 expression by Ca^2+^ is diminished in keratinocytes from IR-knockout mice [48]. In addition, reduction of *KRT5* and *KRT14* expression, with concomitant skin barrier dysfunction, was present in the skin of type 1 diabetic mice [32]. Overall, these observations suggest that the impaired skin function associated with obesity is due, at least in part, to cutaneous insulin resistance and consequent decreases in keratin expression. In addition to their roles in the onset of insulin resistance, inflammatory cytokines and oxidative stress are widely recognized to cause various skin disorders [23, 49, 50]; therefore, obesity-induced inflammatory changes in the skin, as well as those in adipose tissue, appear to be important for obesity-associated skin impairment. Besides oxidative stress and inflammatory cytokines, skin barrier disorder is possibly due to alteration of some other obesity-associated factors. In previous research, adiponectin has been suggested to improve skin barrier function by increasing filaggrin served as natural moisturizing factors [51]. Thus, decreased adiponectin in HFD-fed mice in this study could also impair skin function.

We observed lower expression of Ccnd1, Ccnd2, and Cdk2, and higher expression of Cdkn1a and Cdkn1c in HFD-fed mice than in control mice. Because progression of the cell cycle to the next phase is promoted by CCND1, CCND2, and CDK2, and cell cycle arrest is facilitated by CDKN1A and CDKN1C, which inhibit G1 phase progression and the G1 to S phase transition [40], we speculate that the cell cycle was delayed in the skin of HFD-fed mice. In addition, the mRNA levels of CDKN1A and CDKN1C in cultured keratinocytes increased with an increase in the glucose concentration, although mRNA levels of CCND1, CCND2, and CDK2 were unaffected, consistent with previous reports that high glucose concentrations inhibit keratinocyte proliferation and lead to an imbalance between proliferation and differentiation [32, 52, 53]. These results, together with previous findings, suggest that hyperglycemia associated with obesity suppresses cell cycle progression in the epidermis and may result in skin abnormalities.

In the skin of HFD-fed mice, *AQP3* expression was decreased. Aquaporins play an essential role in maintaining tissue homeostasis by selectively mediating water or glycerol transport [54]. In particular, AQP3 is involved in skin function [37]. Repair of skin barrier function is delayed in AQP3 knockout mice and accelerated in transgenic mice overexpressing AQP3 [55, 56], indicating the involvement of this aquaporin in maintaining the skin barrier. Moreover, administration of glycerol corrects skin defects induced by AQP3 deficiency. These findings, together with the present results, suggest that obesity-associated decreased *AQP3* expression was involved in skin barrier dysfunction via imbalanced glycerol metabolism. In contrast to the expression of keratins and cell cycle regulatory molecules, the expression of AQP3 and desmosomal molecules was not affected by diminished insulin signaling or high glucose treatment *in vitro*. Thus, altered expression of AQP3 and desmosomal molecules in HFD-fed mice appears to be a secondary consequence of other metabolic or biochemical changes associated with obesity.

In summary, we examined the effects of diet-induced obesity on skin function and the mechanisms underlying these effects. We found that skin function was impaired by obesity, at least in part by the induction of cutaneous insulin resistance and hyperglycemia. Our findings indicate that the concept of insulin resistance underlying metabolic diseases such as type 2 diabetes can be extended to the field of skin physiology. Thus, management of obesity and systemic insulin resistance might be beneficial for the amelioration of skin function impairment, as well as obesity-related disorders.

## Supporting information

S1 Table shRNA-encoding DNA sequences. (DOCX)

S2 Table Primers used for RT-qPCR. (DOCX)

S3 Table Organ weights and blood analysis. Mice were fed control or HFD for 26 weeks. On the final day of the experiment, after fasting for 3 h, body weight was measured and blood was collected from the postcaval vein and analyzed using commercially available kits. After blood sampling, liver, muscle (soleus and gastrocnemius), and adipose (epididymal, retroperitoneal, perirenal, and subcutaneous inguinal) tissues were resected and weighed. Values are means ± SD (*n* = 8). *p < 0.05, **p < 0.01, and ***p < 0.001 vs. the Control group (Student *t*-tests). GOT, glutamic oxaloacetic transaminase; GPT, glutamic pyruvic transaminase; HMW, high-molecular-weight; d-ROMs, diacron reactive oxygen metabolites; U.CARR, Carratelli units; BAP, biological antioxidant potential; TAC, total antioxidant capacity; CRE, copper reducing equivalents; TBARS, thiobarbituric acid reactive substances; MDA, malondialdehyde.
(DOCX)

S1 Fig Oral glucose tolerance tests and macrophage infiltration into subcutaneous adipose tissue in mice fed the control or HFD. a-b: After mice were fasted overnight, glucose (2 g/kg body weight) was orally administered, and blood samples were collected from the orbital sinus of alternate eyes at 0, 15, 30, 60, and 120 min. Blood glucose and insulin concentrations were then measured. c: Total RNA was isolated from the subcutaneous inguinal adipose tissue of mice fed control or HFD for 26 weeks, and expression of the genes encoding F4/80 and MCP-1 was measured by RT-qPCR. Expression was normalized to *36B4* and compared to controls. d: F4/80 immunostaining of macrophages in subcutaneous inguinal adipose tissues. Red arrows indicate macrophages infiltrating into adipose tissue. Values are means ± SD (N = 7). **p < 0.01 and ***p < 0.001 vs. the control group (Student’s *t*-test).
(TIF)

S2 Fig Insulin signaling inhibition due to transduction of an IR shRNA viral vector in 3D-keratinocytes. At 1 and 2 days after 3D-keratinocytes were transduced with an IR shRNA viral vector (25 PFU/cell MOI), or incubated with the insulin signaling inhibitor wortmannin (Wort; 2 μM), total protein was extracted from cells and analyzed by western blotting with anti-Akt or anti-phospho-Akt (p-Akt) antibodies.
(TIF)

S3 Fig Alteration of the expression of desmosomal molecules, cell cycle regulatory molecules, and AQP3 due to insulin signaling inhibition in 3D-keratinocytes. Insulin signaling in 3D-keratinocytes was inhibited by addition of wortmannin (Wort; 2 μM) or transduction with an IR shRNA viral vector (25 PFU/cell MOI). Three days after insulin signaling inhibition, the expression of desmosomal molecules, cell cycle regulatory molecules, and AQP3 were analyzed by RT-qPCR (N = 3). Expression was normalized to *36B4* and is shown relative to the control group. Values are means ± SD (N = 3). *p < 0.05 for control vs. Wort and *lac*Z vs. IR shRNA (Student’s *t*-tests).o
(TIF)

S4 Fig Effects of glucose concentration on the expression of keratins, desmosomal molecules, and AQP3. After culture of HEKn cells in growth medium containing 6 (control), 10, 20, or 40 mmol/l glucose for 3 days, the expression of keratins, desmosomal molecules, and AQP3 were analyzed by RT-qPCR. Expression was normalized to *36B4* and is shown relative to the control group. Values are means ± SD (N = 3). ***p < 0.001 vs. the control group (Dunnett’s test).
(TIFF)
