# Supplementary material for: Obesity-associated insulin resistance adversely affects skin function
Source: PLoS One. 2019 Oct 3;14(10):e0223528. doi: 10.1371/journal.pone.0223528 (PMC6776356; doi:10.1371/journal.pone.0223528)
Supplement: S1 Table — (DOCX) [file pone.0223528.s001.docx]

| shRNA name | Sequence (5′ to 3′) |
| --- | --- |
| IR shRNA.1 | GACTCTCAGATCCTGAAGGAGGTGTGCTGTCCCTCCTTCAGGATCTGAGAGTC |
| IR shRNA.2 | GCTGCCACCAGTACGTCATTCGTGTGCTGTCCGAATGACGTACTGGTGGCAGC |
| IR shRNA.3 | GTGACAGACTATTTAGACGTCGTGTGCTGTCCGACGTCTAAATAGTCTGTCAC |
