# Supplementary material for: Obesity-associated insulin resistance adversely affects skin function
Source: PLoS One. 2019 Oct 3;14(10):e0223528. doi: 10.1371/journal.pone.0223528 (PMC6776356; doi:10.1371/journal.pone.0223528)
Supplement: S2 Table — (DOCX) [file pone.0223528.s002.docx]

| Mouse |  |  |
| --- | --- | --- |
| Gene | Forward primer sequence (5′ to 3′) | Reverse primer sequence (5′ to 3′) |
| *36B4* | CTGATCATCCAGCAGGTGTT | CCAGGAAGGCCTTGACCTTT |
| *Krt1* | CAAGTACGAAGAGCTGCAGATC | CGCTGCTCAGCATCATTGA |
| *Krt2* | CCAGCTACAGCAGGATGTCAC | GGAGTTCTTCATACTTGCTGTGG |
| *Krt5* | GAGTACCAGGAGCTCATGAACA | GTAGCCAGAAGAGACACTGTTTG |
| *Krt9* | GGCGAAATCTGATCTGGAGATAC | CACGCATGTCATTGAGGACC |
| *Krt10* | CGACCAATCATCTAAAGGACCAAG | GCCCGTATGAAGAGACTCTTCTA |
| *Krt14* | CAATTCTCCTCTGGCTCTCAGT | GCAGCATGTAGCAGCTTTAGTTC |
| *Krt15* | GGATGCTAAGATGGCTGGTATTG | CTTACGGGAAGAAACCACTTTTCC |
| *Dsg2* | GGAAGCATAGGTCCTCTGCC | GCTAGTTAAGGTCCAGATCCGAC |
| *Dsg3* | GACAGGTGGCTTTATCCCAGTG | GAAGCACCTTCTACCATGGTC |
| *Dsc1* | CTGAAGGACCAGGAGAAGAAGT | GTGGCCTCCTTTGACCATC |
| *Dsc3* | GGGAGGACACATGGATACTGAC | GGAGACCCTCTGCCTTCATAG |
| *Pkp1* | CTGATGTCCAATGGTATGAGTCAG | GCTAGTGAGGAGTCTGGTTACC |
| *Pkp3* | CGTGCTCAACAACCTGGTG | GAGCTTGCTGTACTGCCAG |
| *Pkp4* | GGTGAACATAACCAAAGGCAGAG | GTCTCGCTCTAGCGTGGAC |
| *Jup* | TAAGCGAGTGTCTGTGGAGC | GTCCATGTGCATGTCCAGAG |
| *Dsp* | GTGATTCTGCAAGAGGCTGC | GCCAGTCTTAGCTCCTCTTCC |
| *Aqp3* | GCTCAGAAGTCTTCACGACTG | CCAATCATGAGCTGGTACACG |
| *Ccnd1* | GATGAGAACAAGCAGACCATCC | GTCACACTTGATGACTCTGGAAA |
| *Ccnd2* | GCTTCAGCAGGATGATGAAGTG | GCGTTATGCTGCTCTTGACG |
| *Cdk2* | GCCAGGAGTTACTTCTATGCCT | GCACTGGTTTAGTTACATCCTGG |
| *Cdkn1a* | TCTTGCACTCTGGTGTCTGAG | CTCTTGCAGAAGACCAATCTGC |
| *Cdkn1c* | GAACAAGGCGTCGAACGAC | CCATCTCCGGTTCCTGCTA |
| *Rb* | GGTGTGTAATAGTGACAGAGTGC | CATTCGTGTTCGAGTGGAAGTC |
| *MCP-1* | CTGCTACTCATTCACCAGCAAG | GATCTCATTTGGTTCCGATCCAG |
| *F4/80* | GCCATTGCCCAGATTTTCATCTT | GCTAAGGTCAGTCTTCCTGGT |
|  |  |  |
|  |  |  |
| Human |  |  |
| Gene | Forward primer sequence (5′ to 3′) | Reverse primer sequence (5′ to 3′) |
| *36B4* | CTGGTCATCCAGCAGGTGTT | GCCAAGAAGGCCTTGACCTTT |
| *KRT1* | CAGCATCATTGCTGAGGTCAAG | GATCTAAGTCTCTGGATCACACGA |
| *KRT2* | CATGCAGGATCTTGTGGAGGA | CAATTTCCTGGTTCAGCAGGTC |
| *KRT5* | GACCTGGTGGAAGACTTCAAG | GTGTCAGAGACATGCGTCTG |
| *KRT9* | GGACATTGACAACACTCGCATG | CCAGTCAGCTGACTCATCTCC |
| *KRT10* | TGGAGGCTGACATCAACGG | GGCAGCATTCATTTCCACATTC |
| *KRT14* | GGAACAAGATTCTCACAGCCAC | TCAATCTGCATCTCCAGGTCAG |
| *KRT15* | CAGGCTCAAGTATGAGAATGAGC | GCTGCTGAACTCCTTCATCTC |
| *CCND1* | CAGATCATCCGCAAACACGC | GTCACACTTGATCACTCTGGAGA |
| *CCND2* | GGCAGCTGTCACTCCTCAT | AGCGAGCTCACTTCCTCATC |
| *CDK2* | TGAGATTGACCAGCTCTTCCG | GTCGTAGTGCAGCATTTGCG |
| *CDKN1A* | ACCATGTGGACCTGTCACTG | GAAGATCAGCCGGCGTTTG |
| *CDKN1C* | TCAAGAGAGCGCCGAGCA | GCTGATCTCTTGCGCTTGG |
| *RB* | GTATGTAACAGCGACCGTGTG | GCATTCGTGTTCGAGTAGAAGTC |
