# Supplementary material for: Obesity-associated insulin resistance adversely affects skin function
Source: PLoS One. 2019 Oct 3;14(10):e0223528. doi: 10.1371/journal.pone.0223528 (PMC6776356; doi:10.1371/journal.pone.0223528)
Supplement: S3 Table — Mice were fed control or HFD for 26 weeks. On the final day of the experiment, after fasting for 3 h, body weight was measured and blood was collected from the postcaval vein and analyzed using commercially available kits. After blood sampling, liver, muscle (soleus and gastrocnemius), and adipose (epididymal, retroperitoneal, perirenal, and subcutaneous inguinal) tissues were resected and weighed. Values are means ± SD (n = 8). *p < 0.05, **p < 0.01, and ***p < 0.001 vs. the Control group (Student t-tests). GOT, glutamic oxaloacetic transaminase; GPT, glutamic pyruvic transaminase; HMW, high-molecular-weight; d-ROMs, diacron reactive oxygen metabolites; U.CARR, Carratelli units; BAP, biological antioxidant potential; TAC, total antioxidant capacity; CRE, copper reducing equivalents; TBARS, thiobarbituric acid reactive substances; MDA, malondialdehyde. (DOCX) [file pone.0223528.s003.docx]

|  | Control | | |  | HFD | | |  |  |
| --- | --- | --- | --- | --- | --- | --- | --- | --- | --- |
| Body weight (g) | 42.2 | ± | 3.1 |  | 55.9 | ± | 3.2 |  | ***** |
| Epididymal adipose tissue (g) | 1.23 | ± | 0.32 |  | 1.84 | ± | 0.12 |  | ***** |
| Perirenal adipose tissue (g) | 0.30 | ± | 0.04 |  | 0.88 | ± | 0.20 |  | ***** |
| Retroperitoneal adipose tissue (g) | 0.65 | ± | 0.07 |  | 1.88 | ± | 0.32 |  | ***** |
| Subcutaneous inguinal adipose tissue (g) | 1.36 | ± | 0.23 |  | 2.78 | ± | 0.21 |  | ***** |
| Liver (g) | 1.74 | ± | 0.23 |  | 3.21 | ± | 0.42 |  | ***** |
| Soleus muscle (g) | 0.018 | ± | 0.002 |  | 0.023 | ± | 0.003 |  | **** |
| Gastrocnemius muscle (g) | 0.30 | ± | 0.02 |  | 0.32 | ± | 0.01 |  | **** |
| Triglycerides (mg/dl) | 19.3 | ± | 4.7 |  | 51.2 | ± | 14.3 |  | ***** |
| Total cholesterol (mg/dl) | 139.7 | ± | 15.4 |  | 234.4 | ± | 33.7 |  | ***** |
| LDL cholesterol (mg/dl) | 12.2 | ± | 2.4 |  | 25.9 | ± | 5.7 |  | ***** |
| HDL cholesterol (mg/dl) | 58.3 | ± | 6.1 |  | 82.3 | ± | 11.4 |  | ***** |
| NEFA (mmol/l) | 0.71 | ± | 0.14 |  | 0.83 | ± | 0.12 |  |  |
| GOT (IU/l) | 124.4 | ± | 35.4 |  | 265.1 | ± | 97.7 |  | **** |
| GPT (IU/l) | 82.7 | ± | 43.4 |  | 217.5 | ± | 71.7 |  | ***** |
| Total adiponectin (μg/ml) | 20.1 | ± | 6.1 |  | 14.5 | ± | 1.9 |  | *** |
| HMW adiponectin (μg/ml) | 6.3 | ± | 1.5 |  | 4.2 | ± | 0.7 |  | **** |
| HMW/total adiponectin (%) | 32.0 | ± | 2.9 |  | 29.1 | ± | 1.4 |  | *** |
| TNFα (pg/ml) | 29.3 | ± | 3.2 |  | 27.8 | ± | 1.9 |  |  |
| IL-1α (pg/ml) | 7.6 | ± | 2.9 |  | 12.0 | ± | 5.1 |  |  |
| IL-1β (pg/ml) | 36.8 | ± | 10.8 |  | 58.8 | ± | 15.3 |  | **** |
| d-ROMs (U.CARR) | 154.0 | ± | 35.6 |  | 298.5 | ± | 50.7 |  | ***** |
| BAP (mmol/l) | 2.22 | ± | 0.18 |  | 1.84 | ± | 0.17 |  | ***** |
| BAP/d-ROMs | 15.2 | ± | 4.1 |  | 6.3 | ± | 1.0 |  | ***** |
| TAC (CRE) | 800.1 | ± | 121.4 |  | 593.2 | ± | 76.6 |  | **** |
| TBARS (MDA) (μmol/l) | 4.9 | ± | 1.4 |  | 10.0 | ± | 1.9 |  | ***** |
