# Supplementary figures and images for: Obesity-associated insulin resistance adversely affects skin function
Source: PLoS One. 2019 Oct 3;14(10):e0223528. doi: 10.1371/journal.pone.0223528 (PMC6776356; doi:10.1371/journal.pone.0223528)

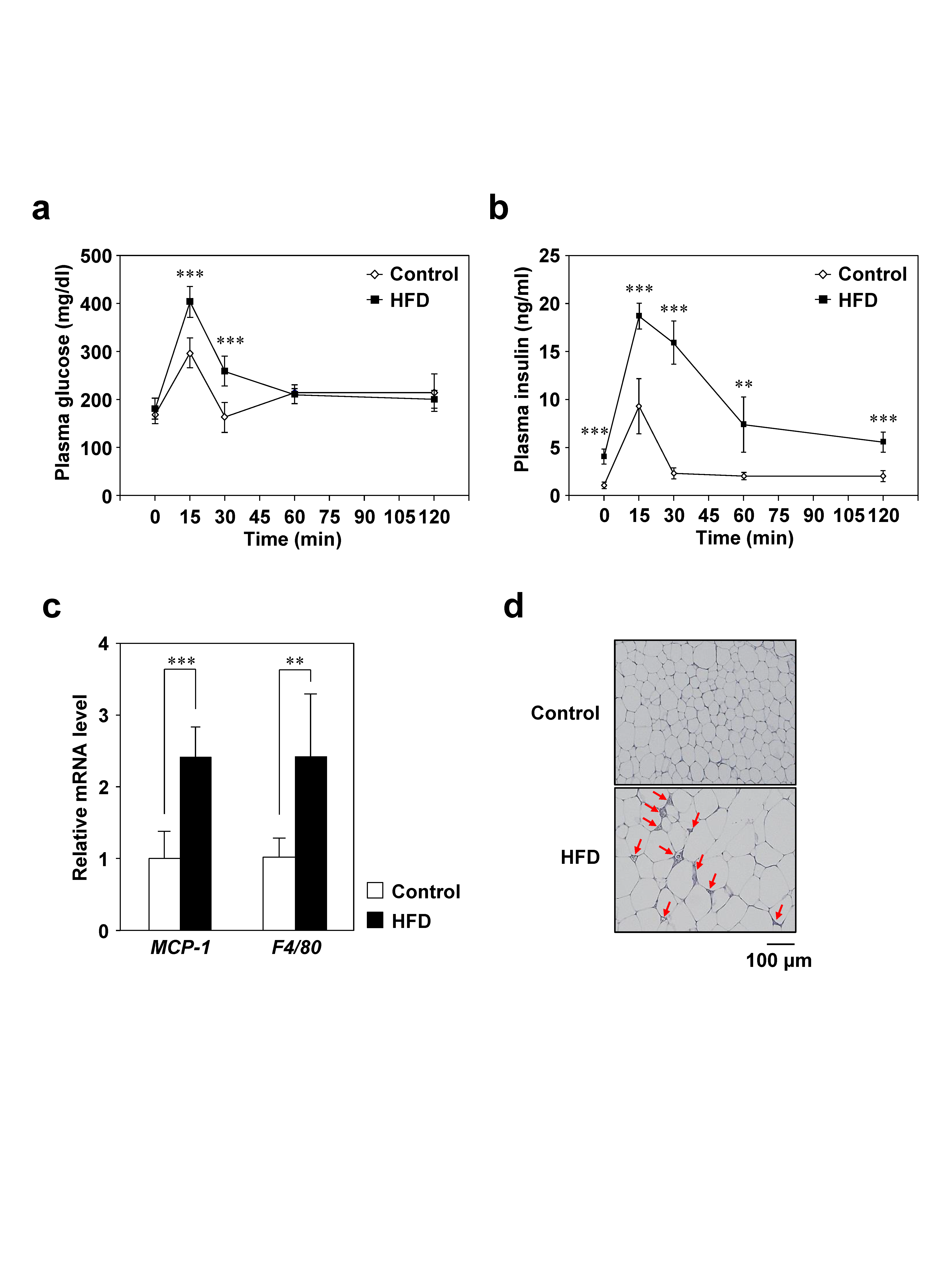

Supplement: S1 Fig — a-b: After mice were fasted overnight, glucose (2 g/kg body weight) was orally administered, and blood samples were collected from the orbital sinus of alternate eyes at 0, 15, 30, 60, and 120 min. Blood glucose and insulin concentrations were then measured. c: Total RNA was isolated from the subcutaneous inguinal adipose tissue of mice fed control or HFD for 26 weeks, and expression of the genes encoding F4/80 and MCP-1 was measured by RT-qPCR. Expression was normalized to 36B4 and compared to controls. d: F4/80 immunostaining of macrophages in subcutaneous inguinal adipose tissues. Red arrows indicate macrophages infiltrating into adipose tissue. Values are means ± SD (N = 7). **p < 0.01 and ***p < 0.001 vs. the control group (Student’s t-test). (TIF) [file pone.0223528.s004.tif]

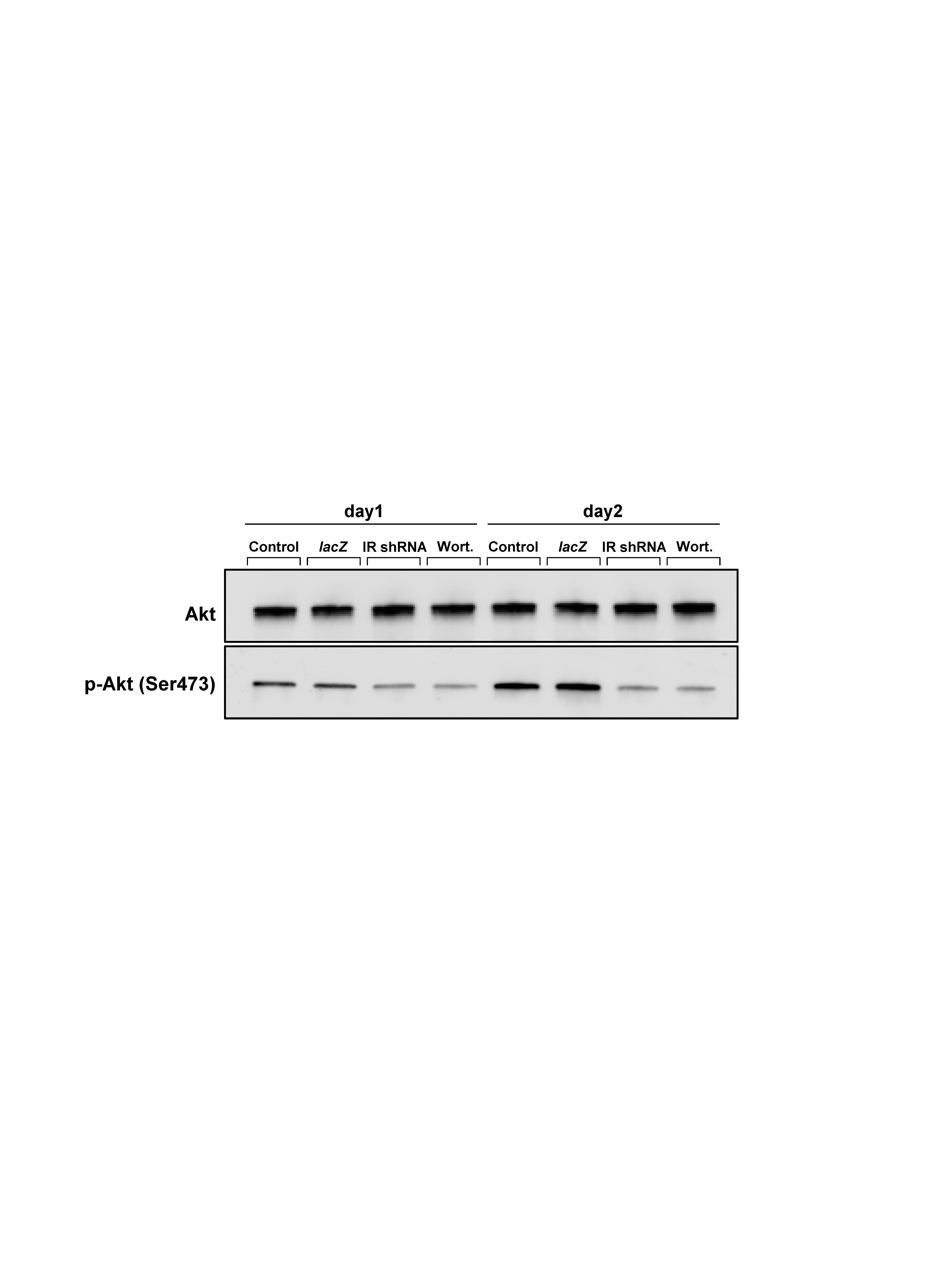

Supplement: S2 Fig — At 1 and 2 days after 3D-keratinocytes were transduced with an IR shRNA viral vector (25 PFU/cell MOI), or incubated with the insulin signaling inhibitor wortmannin (Wort; 2 μM), total protein was extracted from cells and analyzed by western blotting with anti-Akt or anti-phospho-Akt (p-Akt) antibodies. (TIF) [file pone.0223528.s005.tif]

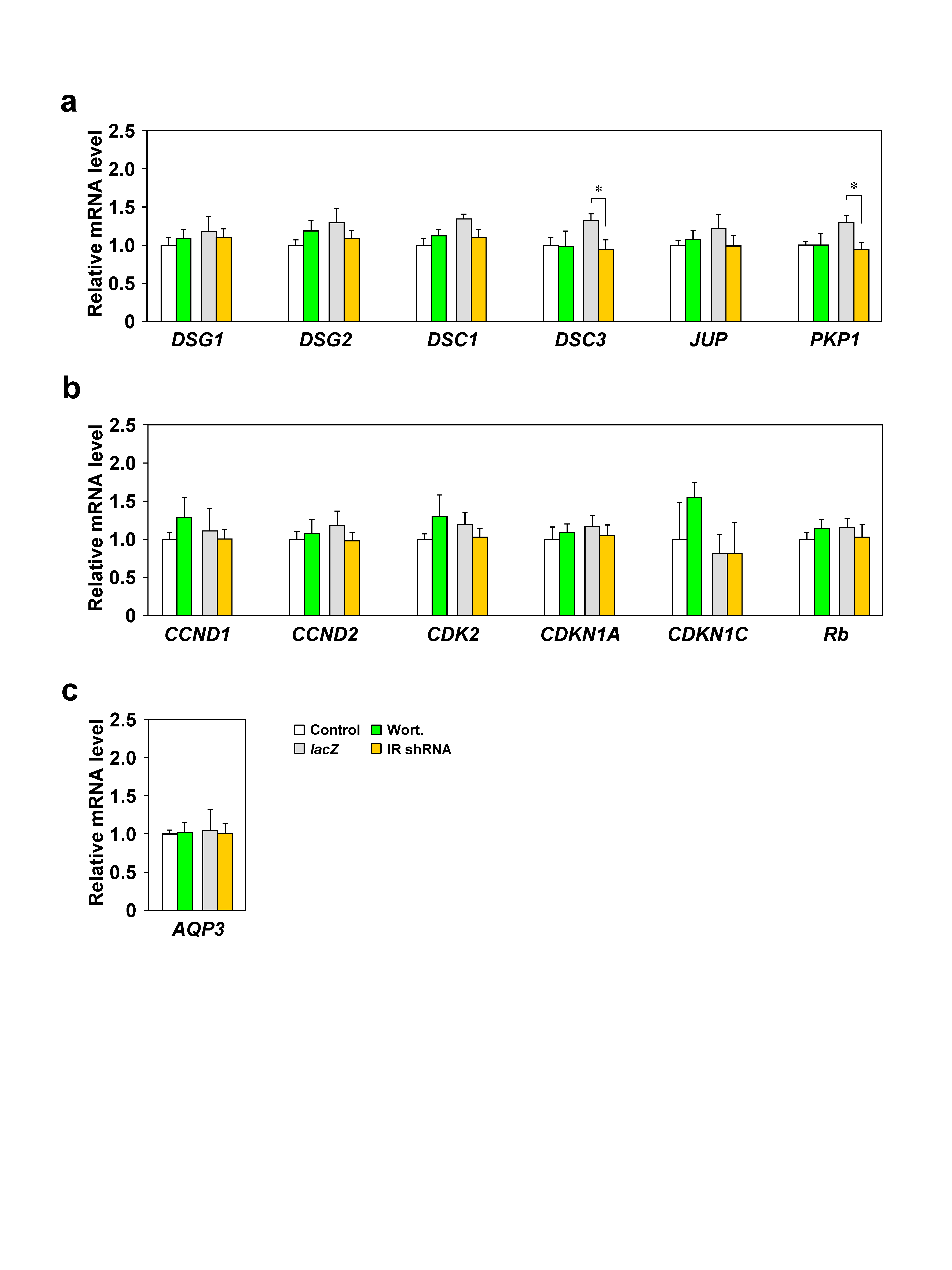

Supplement: S3 Fig — Insulin signaling in 3D-keratinocytes was inhibited by addition of wortmannin (Wort; 2 μM) or transduction with an IR shRNA viral vector (25 PFU/cell MOI). Three days after insulin signaling inhibition, the expression of desmosomal molecules, cell cycle regulatory molecules, and AQP3 were analyzed by RT-qPCR (N = 3). Expression was normalized to 36B4 and is shown relative to the control group. Values are means ± SD (N = 3). *p < 0.05 for control vs. Wort and lacZ vs. IR shRNA (Student’s t-tests).o (TIF) [file pone.0223528.s006.tif]

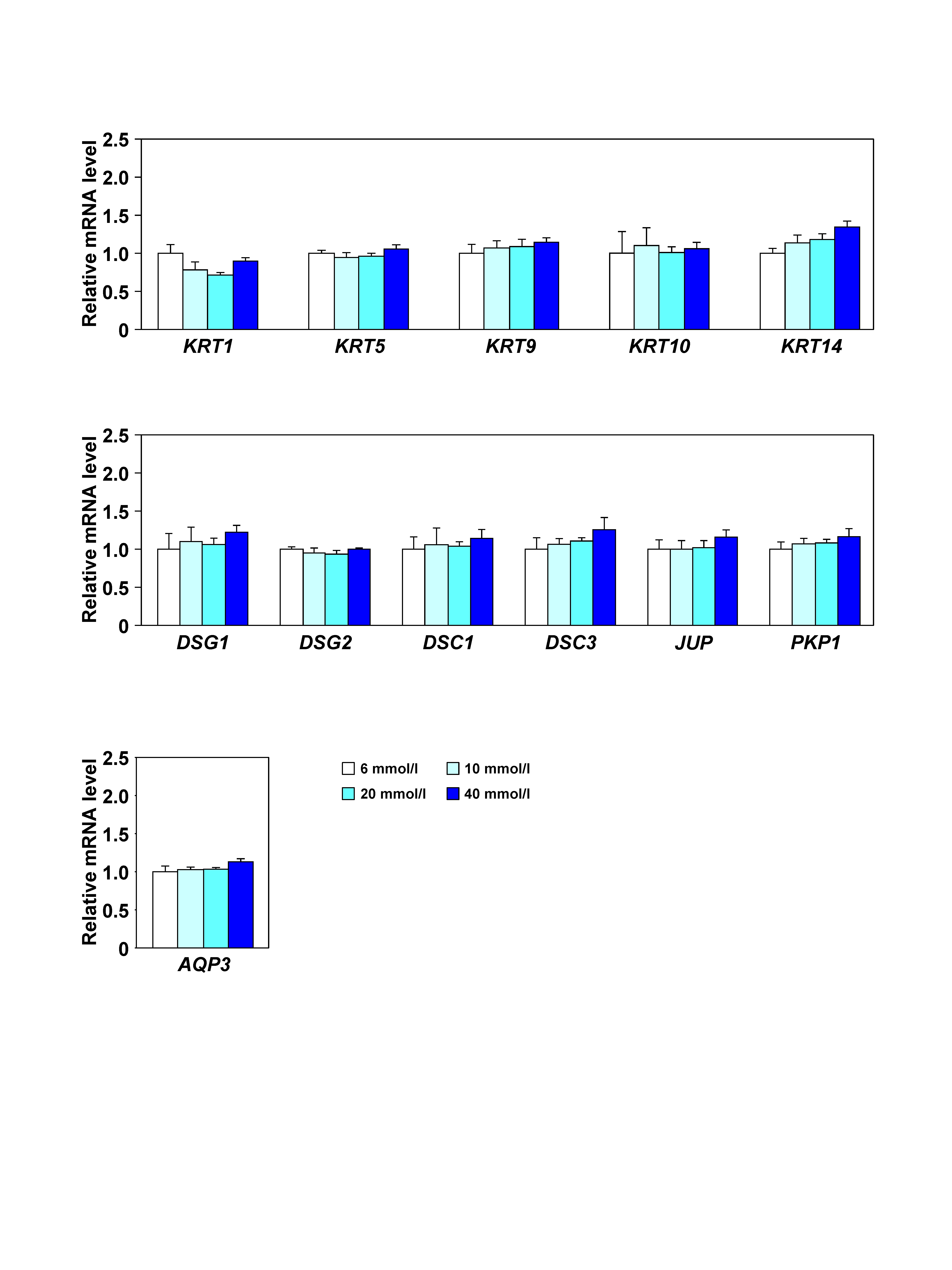

Supplement: S4 Fig — After culture of HEKn cells in growth medium containing 6 (control), 10, 20, or 40 mmol/l glucose for 3 days, the expression of keratins, desmosomal molecules, and AQP3 were analyzed by RT-qPCR. Expression was normalized to 36B4 and is shown relative to the control group. Values are means ± SD (N = 3). ***p < 0.001 vs. the control group (Dunnett’s test). (TIFF) [file pone.0223528.s007.tiff]
